# Has COVID-19 Changed the Lifestyle and Dietary Habits in the Spanish Population after Confinement?

**DOI:** 10.3390/foods10102443

**Published:** 2021-10-14

**Authors:** Carmen Álvarez-Gómez, Magdalena De La Higuera, Lorenzo Rivas-García, Javier Diaz-Castro, Jorge Moreno-Fernandez, Magdalena Lopez-Frias

**Affiliations:** 1 Nephrology Service, University Hospital Clínico San Cecilio, 18016 Granada, Spain; Carmen.alv.g@gmail.com; 2 Department of Endocrinology and Nutrition, Clínica Universidad de Navarra, 28027 Madrid, Spain; mhiguera@unav.es; 3 Department of Physiology, University of Granada, 18071 Granada, Spain; lorenrivas@ugr.es (L.R.-G.); maglopez@ugr.es (M.L.-F.); 4 Institute of Nutrition and Food Technology “José Mataix Verdú”, University of Granada, 18071 Granada, Spain; 5 Biomedical Research Centre, University of Granada, 18016 Armilla, Spain; 6 Instituto de Investigación Biosanitaria IBS, 18016 Granada, Spain

**Keywords:** COVID-19, dietary habits, lockdown, post-confinement, lifestyle, e-survey, Mediterranean diet adherence, Spanish population

## Abstract

Since 2020, the world has been immersed in a change in lifestyle (social, lifestyle, nutri-tion and physical activity patterns), due to the appearance of COVID-19 and the strict mobility measures which were implemented to prevent its spread. All these changes had a nutritional impact on people, modifying their dietary guidelines. This cross-sectional study was carried out to assess whether dietary habits, lifestyle, and adherence to the Mediterranean diet among the Spanish adult population (25–65 years old) during confinement was modified during the post-confinement period, using an e-survey through social networks, involving 510 subjects. A decrease in the intake of ultra-processed products, with a correlation between weekly food delivery orders at home and the consumption of salty snacks, sugary drinks, and processed pastries was also recorded. Most of the subjects performed physical exercise on a regular basis, maintaining the body weight in half of the participants. During the post-confinement period a substantial proportion of the population had healthy lifestyle and dietary habits, including the adequate consumption of fruits, vegetables and legumes; adequate time was spent preparing meals and the population did not regularly order food at home, which in the long term, reduced the risk of several diseases.

## 1. Introduction

The rapid spread of SARS-CoV-2, commonly known as the coronavirus which causes the disease called COVID-19, consists of an acute respiratory syndrome whose most characteristic clinical symptom is bilateral pneumonia, and has led to numerous changes at a global level. Since its appearance in the Chinese city of Wuhan in November 2019, the virus displayed a rapid progressive worldwide expansion, until it was considered a pandemic by the World Health Organization on 11 March 2020 [1]. The first case of infection by this virus reported in Spain occurred in January 2020 in the Canary Islands and in February of that same year in the peninsula, progressively showing an increase in cases throughout the national territory, causing a collapse of the health system and the implementation of a necessary state of alarm in Spain, which came into force on 14 March 2020. This meant that the population remained in home confinement except for carrying out essential activities as a measure to contain the spread of the virus, a situation that was extended until the beginning of May 2020. From this time, people were allowed to go out into the street within a set time according to their age groups. This continued until it was gradually possible to extend the closing and opening hours of non-essential businesses and shops, for which timetables for social mixing in establishments, as well as border controls, were adapted according to the accumulated incidence in each region of the national territory. On 9 May 2020, the state of alarm finished in Spain, allowing free movement throughout the country [2].

This exceptional situation has been studied due to the great impact it caused at the social and health level. With regard to food consumption, a global change was observed in dietary habits due to the initial panic buying due to the lack of available food [3], as well as the consumption of processed products or foods rich in carbohydrates, because these could increase the production of serotonin and prevent depression and mood swings. Additionally, in states of low physical activity there is a tendency towards the intake of this group of foods [4], leading to a worsening in the dietary habits of the different populations studied [5,6,7]. The changes in physical activity worldwide, due to the need to implement total quarantines in several countries, was detrimental to an increase in a sedentary lifestyle due to the inability to perform physical activity in open spaces and the lack of areas at home suitable for physical activity [8]. All these changes caused alterations in mental health [9], and a loss of muscle mass associated with an increase in body fat and weight gain, in addition to promoting other chronic diseases such as diabetes and cardiovascular disease, which were a significant public health issue, due to the morbidity and mortality risks involved [10,11]. It should also be taken into account that cardiovascular disease, diabetes and excess body fat are associated with a greater risk of COVID-19 infection and more serious symptoms, underlining the importance of avoiding the development of such morbidities [12].

Due to this health problem, a series of tips on how to lead a healthy lifestyle were published, agreeing on the recommendation to prioritize the basic nutritional elements of the Mediterranean diet (fruits and vegetables, cereals, polyunsaturated fats, etc.), in addition to avoiding distractions while eating meals, establishing the most convenient time to eat them and performing physical activity on a daily basis to avoid health problems associated with a sedentary lifestyle [13,14,15].

When all these data were analyzed in Spain, the scientific literature concluded that there was an improvement in the diet quality and, in this sense, a reduction in the consumption of red meat and processed foods, together with an increase in the consumption of fruits and vegetables and a greater adherence to the Mediterranean diet, was found during the confinement [16]. However, these results were in contrast to those reported by other studies, which determined that the Spanish diet during confinement was of a lower quality and did not follow the ideal pattern of the Mediterranean diet. Instead, the Spanish diet during confinement was more caloric and had an increased intake of red meat [17] and foods with a high sugar content, due to stress [18].

With respect to the physical activity performance in the Spanish population during confinement, a decrease was observed, with an increase in a sedentary lifestyle of up to 23.8%, due to the limitation of outdoor physical activity [19].

This study aims to analyze whether the hygiene and dietary habits practiced during confinement in Spain continued during the post-confinement period, from May 2020. For this purpose, a survey consisting of 36 items was carried out where socio-demographic aspects, general nutrition and the intake of different food groups, physical activity and toxic habits were assessed, aimed at the Spanish active population.

## 2. Materials and Methods

### 2.1. Study and Survey Methodology

This cross-sectional study was carried out on Spanish adults (25–65 years old). Participation in the study was completely voluntary and anonymous. The survey was conducted in agreement with the Declaration of Helsinki [20], and all data were collected and recorded according to the Spanish Data Protection Law (Organic Law 3/2018) in line with European Commission General Data Protection Regulation (679/2016). The survey was designed to collect information regarding social characterization, nutritional behavior and healthy lifestyles of people who resided in Spain before the state of alarm decreed in the country (RD 463/2020, 14 March 2020), when face-to-face work activities were restricted to only basic and essential services. The information asked included several questions which could help to understand whether the habits related to healthy lifestyle were modified by the unprecedented COVID-19 pandemic situation during the post-confinement period. This was a study with sampling based on an online survey (Supplementary Material Table S1) using the Google Forms web survey platform (Google Inc.; Santa Monica, CA, USA), shared and spread via email and common social media networks, including Facebook (Facebook, Inc., Menlo Park, CA, USA), WhatsApp (WhatsApp LLC, Menlo Park, CA, USA), Twitter (Twitter, Inc., San Francisco, CA, USA) and Instagram (Facebook, Inc., CA, USA). The surveys were collected from 15 March to 4 April 2021 (during the post-confinement period) and we obtained a sample of 510 subjects. At the end of the online questionnaire, each participant provided informed consent. In addition, the adherence to a Mediterranean diet was assessed with a questionnaire including several items. This was useful for a short and simple assessment of the healthiness of dietary patterns in Mediterranean populations and reduced the costs and data collection time [21].

### 2.2. Sociodemographic Parameters

This section included eight items which described and evaluated the population regarding their gender, age, and job conditions. Moreover, the section also included some questions about eating habits such as eating times, mode of eating (eating alone, watching TV/using PC) and the times that the participants ordered food delivery.

### 2.3. Nutritional Habits

We included 17 questions related to three dimensions: the habit of eating some essential Mediterranean diet foods, such as bread and olive oil, daily and the type of this food that was consumed, in order to make a comparison with the traditional pattern of Mediterranean diet. Other sections included the servings of different type of foods that were associated with the Mediterranean diet (legume, milk, fruits), as well as food that was not included in this diet, such as snacks or industrial bakery goods.

### 2.4. Physical Activity and Habits

This section included the habit of practicing sport and some characteristics such as the time, activity, or place where the sport sessions took place. Furthermore, we included some harmful lifestyle habits, such as drinking alcohol and smoking, and the impact of post-confinement on these habits. Finally, we asked about the participants’ perceptions Mediterranean diet.

### 2.5. Statistical Analysis

Descriptive parameters were obtained by the codification of the variable studied. Pearson tests were employed to evaluate the correlation between the variables. All the analyses were performed using SPSS 20.0 SPSS (SPSS, Chicago, IL, USA). Differences were considered statistically significant at a probability level < 5%.

## 3. Results

### 3.1. Socio-Demographic Registry

Regarding participation according to sex, there was a predominance of women (74.5%) compared to men (25.3%). When analyzed by age group, a fairly equal participation was observed among the four groups, being slightly lower in the ages between 25 and 34 years.

With regard to the domicile, 76.9% of those surveyed lived with a family, followed by 11% who shared a residence. In response to the work modality among those surveyed, it was observed that face-to-face and blended attendance predominated over the rest of the modalities (57.1% and 17.8%, respectively). The socio-demographic parameters of the population studied are listed in Table 1.

### 3.2. Nutritional Habits

Table 2 summarizes the nutritional habits of the population studied. When asked how many meals a day they consumed, the majority (88.2%) consumed between 3 and 5 meals. When asked how these meals were made, 75.9% reported that they ate them in company, followed by 13.3% who usually ate while watching TV or using the computer.

When analyzing the time devoted to meals, the populations were divided between 30 min–1 h and less than 30 min (51% and 48.2%, respectively). When asked about food intake, the majority (68.8% of respondents) did not order delivery food at home, while 27.6% of the individuals analyzed made delivery food orders between 1 and 3 times per week.

About the daily consumption of bread, 78.7% reported a daily consumption and when the type of bread consumed was analyzed, it was observed that 51% of the bread consumed was white wheat bread, followed by whole wheat bread (35.7%).

With regard to oil consumption, the vast majority of the respondents reported a daily consumption (96.9%), being the predominant olive oil (98.2%).

Regarding the daily servings of fruits and vegetables consumed, the majority of the subjects surveyed consumed two servings from both food groups (35.7% and 31.2%, respectively).

Of those surveyed, 40.4% reported the intake of two servings of dairy products per day, followed by 31.2% who consumed a single daily serving, with the percentage of those who did not consume any serving of these products being the lowest (3.9%).

Table 3 shows the food consumption patterns of the population. When analyzing the daily intake of nuts, 52% of the subjects surveyed reported a daily intake, while 31% did not consume these foods on a regular basis.

With regard to legumes, 37.3% of those surveyed reported the consumption of these twice a week, while 30% only consumed them once a week.

The vast majority of respondents consumed between two and three servings of potatoes, rice or pasta on a weekly basis (29.4% and 26.7%, respectively).

Regarding the intake of eggs, 30.8% of those surveyed mentioned an intake of twice a week, followed by 29.5% who had an intake three times a week.

When analyzing the weekly consumption of the rest of the food groups, a low consumption of vegetable drinks was observed (78.3% of those surveyed denied consuming these drinks), a moderate consumption of red meat and blue fish was recorded (49.3% and 51.5% reported its consumption between one and two times a week) and a significant consumption of white meat was recorded, since 64% of those surveyed reported that they consumed white meat three or more times a week.

Regarding the consumption of processed foods, we could observe that the vast majority of our study population denied the intake of sugary drinks per week (55.5%), 38.2% reported that they did not consume salty snacks on a weekly basis, followed by 28.8% who consumed them once a week. In addition, with respect to processed pastries, it was observed that 52.9% of those surveyed denied their weekly consumption.

Regarding the adherence to the Mediterranean diet), in most of the subjects surveyed, their intake pattern was adapted to the Mediterranean diet, with 43.7% of the responses valuing it as remarkable and 27.8% as outstanding.

### 3.3. Physical Activity and Body Weight

In the section of the questionnaire where the physical activity performed by the subjects was assessed, 81% answered affirmatively to the question of performing some activity. With regard to where this activity was practiced, the majority of those surveyed performed such activity outdoors (61.4%), and the percentage of those who performed physical activity in a sports center or at home was very similar (22.9% and 23.1%, respectively). Most of those surveyed performed physical activity between two and three days per week (19.2% and 19.4%). Finally, 42.7% performed between 30 min and one hour of physical activity. In the final part of the questionnaire, the participants were asked if they had noticed variations in body weight with respect to confinement. The most common answer was that the body weight remained unchanged (52.9%), followed by those who recorded an increase in body weight (32.4%) (Table 4).

### 3.4. Toxic Habits

In the toxic habits section, 85.7% of the subjects reported that they did not smoke. Likewise, 10.8% of smokers reported that their tobacco consumption remained the same with respect to the confinement. With regard to alcohol, the majority reported that they consumed it (62.4% of those surveyed). As with tobacco consumption, alcohol consumption remained the same with respect to the confinement in 35.9% of those surveyed (Table 5).

Finally, with regard to the time devoted to meals, we found that as the age of the subjects increased, the time devoted to meals also increased (*p* < 0.01). In addition, when the time spent eating was longer, the subjects also consumed more vegetables and fruits (*p* < 0.05). In the analysis of the consumption of food at home, it was observed that when the number of food delivery orders at home was higher, the consumption of sugary drinks (*p* < 0.01), salty snacks (*p* < 0.01) and pastries (*p* < 0.05) also increased (Table 6).

## 4. Discussion

The COVID-19 pandemic caused a great change in the daily lives of people worldwide, as was reflected in different articles on the changes in nutritional guidelines, mental health and physical activity, among other aspects, from numerous studies carried out at a global level. These studies included those carried out by Pérez Rodrigo et al. [19], who evaluated nutritional changes in relation to confinement in Spain; Sidor et al. [5], who reported the impact of the COVID-19 pandemic on the food consumption habits of the Polish population; Alhusseini et al. [6], who evaluated the effects of the pandemic on nutritional patterns in Saudi Arabia; and Izzo et al. [7], who reflected on the impact of confinement on nutritional habits in Italy.

In the current study, we analyzed whether the changes observed in dietary and lifestyle habits during the period of confinement in the adult Spanish population were maintained after the return to normality in the post-confinement period (face-to-face work, the return of performing physical exercise outdoors, and a greater accessibility to fresh foods in local markets, among others).

A balanced participation was observed with respect to the different age groups, but not with respect to gender, since there was a greater female participation, results which coincided with those found by Rodríguez Pérez et al. who reported a greater collaboration in the questionnaires carried out for women [13]. These results are particularly interesting because a previous study found that women reported eating more unhealthy foods [22]; however, the current study revealed very healthy patterns of dietary habits and lifestyle habits in all the subjects surveyed. In addition, our results were in accordance with previous studies, which showed that, in Spain today, women carry out most of the food shopping and are the main players involved in food-related activities [23].

With regard to housing, the majority of the surveyed population lived in a family group which, according to Rodríguez Pérez et al. [13], was a positive factor in adhering to a Mediterranean diet. In our work, the self-perception of the majority of the surveyed subjects was that their intake pattern was adapted to the Mediterranean diet. Likewise, this positive impact of the family group was reflected in the majority of the subjects, who usually ate meals in company. Our study showed that the degree of adherence to the Mediterranean diet was higher than that which was evidenced in other studies [13,24].

On the other hand, the vast majority of the subjects ate between 3 and 5 meals a day, and half of them spent between 30 min and 1 h consuming these meals. A statistically significant correlation was found between age and the time devoted to meals: as the age increased, more time was devoted to the meals. Most of the subjects did not order food at home in the post-confinement stage, implying that there was an entrenched habit in our population of cooking at home, a practice which increased in frequency during confinement in Spain in the first wave of the pandemic, in accordance with a finding previously reported by Pérez Rodrigo et al. [19], who showed an increase of 14.1% in the time spent at home during confinement. In our study, correlations were found between the ordering of food at home and the consumption of ultra-processed foods, since the greater the number of orders of this type of food the greater the consumption of salty snacks, processed pastries, and sugary drinks.

The vast majority of the subjects had a face-to-face work modality, followed by the blended work modality. This could have a positive influence when it came to reducing the possible intake of hypercaloric foods because the prolonged stay at home could lead to anxiety and a lack of routine and, consequently, the increased consumption of this type of food as a way to relieve stress, as reflected in the studies by Savarese et al. [3], Muscogiuri et al. [4] and Berengui et al. [18]. However, on the other hand, returning to face-to-face work prevented dedicating the recommended time to meals and their preparation processes; therefore, people consumed precooked and processed foods with scarce nutritional value.

When we analyzed the data obtained from our survey regarding the nutritional habits in the post-confinement period, a general trend was observed towards a consumption of fruits and vegetables within the appropriate parameters according to the nutritional recommendations. As previously reported [19], this positive habit improved during the confinement in Spain, increasing the consumption of fruit and vegetables by 27% and 21%, respectively, compared to the pre-pandemic situation.

With regard to the intake of legumes, our results were similar to those reported by Pérez Rodrigo et al. [19], revealing an increase in this food consumption in a quarter of the population during confinement. In the current study, a positive correlation was found between the time devoted to meals and the intake of these foods, since the longer preparation times of meals caused a greater consumption of fruits, vegetables and legumes.

In relation to the intake of dairy products, in Spain it was observed that, during confinement, almost 90% of individuals habitually consumed 12 weekly servings of these foods on average [19]. Our study presented very similar data, since the majority of those surveyed reported the intake of at least one dairy product per day; almost half of the subjects surveyed consumed two daily servings, with an average of 12 weekly servings of dairy products.

Egg intake increased considerably during confinement, being estimated at around 25%, but this increase was not observed homogeneously among all the age groups, but was more evident among subjects between 18 and 34 years [19]. In our study, carried out in the post-confinement period, most of those surveyed reported an egg intake of two to three times a week; therefore, we did not find any correlation between age and the intake of this food.

The consumption of red meat reduced during the period of confinement, one of the reasons for this being the difficulty in acquiring this type of food due to the influx in purchases that were made in the weeks prior to the state of alarm, as previously reported [13]. However, in the current study, the consumption of this type of meat was observed from once to twice a week in half of the subjects, due to the access to local markets which opened due to the return of freedom of movement within the country. 

As mentioned above, several studies worldwide, such as those by Savarese et al. [3], Muscogiuri et al. [4], and Berengui et al. [18], reported a greater intake of processed or hypercaloric products caused by the stress of confinement due to the COVID-19 pandemic. In addition, an international online study, with the majority of participants being women, also showed that the number of snacks between meals increased significantly during home confinement [25]; however, in our study, during post-confinement, it was observed that, regarding the intake of salty snacks, processed pastries and soft drinks on a weekly basis, the majority response was that these types of products were not consumed. These results were similar to those reported by Pérez Rodrigo et al. [19]; although, in the study by Rodríguez Pérez et al. [13], 39% consumed this type of food one to three times a week and that 37% consumed it sporadically. 

The data obtained in the current study regarding physical activity after confinement indicated that most of the subjects practiced some degree of activity, exercising between two and three days a week. These data differ from the studies on physical activity carried out during quarantine, in which a great reduction in physical activity was observed in Spain; it was the European country with the highest rate of inactivity (close to 38%) as reported previously [26], since during the entire confinement there was no space provided at home to carry out exercise. This could lead to a loss of muscle mass and weight gain, which were factors associated with a worse prognosis in cases of SARS-CoV-2 infection by hindering the immune response to the disease [27].

When analyzing the harmful habits, the vast majority of those surveyed were non-smokers and, for those who did smoke, the level of smoking remained similar during confinement; these results were in agreement with a previous study [28]. With respect to alcohol, more than the half of the subjects reported that they consumed it; however, among the consumers, a third of the subjects did not report variations in their consumption in the post-confinement period compared to the confinement period, followed by a decrease in alcohol consumption in a quarter of the population. Alcohol consumption decreased compared to the previous year during the period of confinement in Spain [13]. However, previous studies reported that, in times of crisis, alcohol consumption increases among people who are less likely to acquire the drugs they habitually consume, which is why they consume alcohol; to replace or alleviate the difficulties drug withdrawal entails [29].

In relation to body weight, half of the subjects reported that they maintained their weight and a third of the subjects reported an increase in body weight. In studies carried out during confinement in the Spanish population, Battle et al. described a higher caloric intake with a lower nutritional quality, estimating a 6% increase in caloric intake in relation to pre-pandemic trends [16], and the survey carried out by the Spanish Society for the Study of Obesity reported a weight gain in 49.8% of the respondents, which ranged between 1 and 3 kg, attributed to a higher dietary caloric intake and, above all, to a reduction in physical activity [30]. However, Pérez Rodrigo et al. [19] observed a trend towards a greater consumption of healthy foods, with less consumption of foods of less nutritional interest, and an increase in the practice of cooking at home. This change towards healthier dietary patterns was also observed by Rodriguez Pérez et al., as mentioned previously. Therefore, although changes in eating habits and physical exercise were difficult to maintain in the long term, in our population studied during post-lockdown we observed the maintenance of this healthier dietary pattern [13]. 

Although these data could not be extrapolated to the entire Spanish population as it was a convenience sample, they coincided with the data published by the Spanish Ministry of Agriculture, Fisheries and Food on the consumption habits of Spanish households during the month of March 2020, which showed a greater presence of vegetables, legumes, rice or dairy products in the shopping basket, as well as a greater demand for fish and fishery products [31].

Finally, the questionnaire was a valid and suitable tool to assess the dietary and lifestyle changes, although this study has certain limitations, including its cross-sectional design which prevented us from observing causality, as well as the use of the online survey which could have underreported certain aspects such as alcohol or tobacco consumption. Moreover, further questionnaires would be helpful to assess dietary habit changes during the post-confinement period, in order to provide dietary advice for the Spanish population.

## 5. Conclusions

The current study gave us information on how the lifestyle and dietary habits changed during the post-confinement period in the Spanish adult population. The data reported in the current study suggested that, during the post-confinement period, a substantial proportion of the population had healthy lifestyles and dietary habits, including the adequate consumption of fruits, vegetables and legumes, an adequate time spent preparing meals and the infrequent ordering of food at home. In addition, in the post-confinement period, most of the subjects performed physical exercise on a regular basis which, in the long term, could reduce the risk of several diseases (obesity, diabetes, cardiovascular diseases), taking into account that during confinement dietary and lifestyle habits were very limited due to the restriction of movements, which implied a lack of physical activity and access to local markets to obtain fresh foods. It would be desirable if the improvement in the lifestyle habits observed during the post-confinement period could be maintained in the long term as they would have a positive impact on the health of the Spanish population.

## Figures and Tables

**Table 1:** Socio-demographic parameters (values are expressed as percentage).

Age (%)	25–34 years 22	35–44 years 24.9	45–55 years 26.5	55–64 years 26.6		
Gender (%)	Male	Female				
	25.3	74.7				
Residence type (%)	Living alone	Shared house	Family home	Collective residence		
	9.3	11.1	76.5	3.1		
Work type (%)	Face-to-face	Blended work	Online	Unemployed	Students	Others
	57	17.9	8	5.8	4.7	6.6
Frequency of eating per day (%)	<3	3–5	>5			
	10.1	88.3	1.6			
Mode of eating (%)	Alone	With family/friends	While watching TV/using PC	Others		
	8.6	76.1	13.2	2.1		
Time for eating (%)	<30 min	30–60 min	>60 min			
	48.2	51	0.8			
Food delivery (%)	0 days per week	1–3 days per week	4–6 days per week	>6 days per week		
	68.7	27.8	0.8	2.7		

**Table 2:** Nutritional habits (values are expressed as percentage).

Do you eat bread daily? (%)	Yes	No						
	78.8	21.2						
Type of bread (%)	Wheat bread (white wheat flour)	Whole wheat bread (white whole wheat flour)	Sandwhi ch bread	Milk bread				
	51	35.7	12.0	1.3				
Do you eat oil daily? (%)	Yes	No						
	96.9	3.1						
Type of oil (%)	Extra Virgin olive oil	Sunflower oil	Coconut oil					
	98.2	1.2	0.6					
Vegetables (%)	0 serving per day	1 serving per day	2 servings per day	3 servings per day	4 servings per day	5 servings per day		
	3.7	29.2	35.8	20	6.6	4.7		
Fruit (%)	0 serving per day	1 serving per day	2 servings per day	3 servings per day	4 servings per day	5 servings per day		
	8	27.6	31.1	21.2	6.6	5.5		
Milk/Dairy (%)	0 serving per day	1 serving per day	2 servings per day	3 servings per day	4 servings per day	5 servings per day		
	3.9	31.5	40.3	15.4	5.6	3.3		
Dried fruits (%)	0 serving per day	1 serving per day	2 servings per day	3 servings per day	4 servings per day	5 servings per day		
	31.1	51.8	11.9	3.2	0.8	1.2		
Legume (%)	0 serving per week	1 serving per week	2 servings per week	3 servings per week	4 servings per week	5 servings per week	6 servings per week	7 servings per week
	4.3	30	37.2	18.9	4.7	3.3	0.4	1.2
Potatoes, pasta, rice (%)	0 serving per week	1 serving per week	2 servings per week	3 servings per week	4 servings per week	5 servings per week	6 servings per week	7 servings per week
	1.9	14.4	29.4	26.7	12.3	6.8	3.3	5.2
Eggs serving (%)	0 Unit per week	1 Unit per week	2 Units per week	3 Units per week	4 Units per week	5 Units per week	6 Units per week	7 Units per week
	1.8	11.9	31.2	29.4	12.9	6.8	3.3	2.7
Sugary drinks (%)	0 Unit per week	1 Unit per week	2 Units per week	3 Units per week	4 Units per week	5 Units per week	6 Units per week	≥ 7 Units per week
	55.3	18.7	8.6	6.6	2.3	2.9	2.3	3.3
Snacks (%)	0 Unit per week	1 Unit per week	2 Units per week	3 Units per week	4 Units per week	5 Units per week	6 Units per week	≥ 7 Units per week
	37.9	29	17.1	8.9	2.5	1	1.6	2
Industrial bakery (%)	0 Unit per week	1 Unit per week	2 Units per week	3 Units per week	4 Units per week	5 Units per week	6 Units per week	≥ 7 Units per week
	53.1	23.9	9.7	5.4	3.5	2.1	0.4	1.9

**Table 3:** Weekly food consumption (values are expressed as percentage).

	None Consumed in a Week
Vegetable drinks	78.4
Red meat	32.5
White meat	4.4
Sausage meat	32.9
White fish	13.9
Blue fish	20
	Consumed 1–2 serving/week
Vegetable drinks	8.6
Red meat	49.5
White meat	62
Sausage meat	38.7
White fish	59.9
Blue fish	51.5
	Consumed ≥ 3 serving/week
Vegetable drinks	12.1
Red meat	15
White meat	63.8
Sausage meat	33.1
White fish	25.1
Blue fish	12.9

**Table 4:** Physical activity (values are expressed as percentage).

Do you practice sport? (%)	Yes	No						
	81.1	18.9						
Where do you practice sport? (%)	Home	Outdoor	Sport center					
	23.5	61.3	15.2					
How many days practice sport (days/week)? (%)	0 day per week	1 day per week	2 days per week	3 days per week	4 days per week	5 days per week	6 days per week	7 days per week
	15.9	6.6	19.3	19.3	14.9	13	6.5	4.5
Time session (%)	0 min	<30 min	30–60 min	>60 min				
	15.6	7.3	42.6	34.6				

**Table 5:** Toxic habits (values are expressed as percentage).

Do you usually smoke? (%)	Yes	No		
	14.2	85.8		
Did you increase/decrease smoking after the confinement? (%)	No	Increased	Equal	Decreased
	79.8	4.9	10.7	4.6
Do you usually drink alcohol? (%)	Yes	No		
	62.6	37.4		
Did you increase/decrease drinking alcohol after the confinement? (%)	No	Increase	Equal	Decrease
	28.3	10.6	35.8	25.3
Do you consider that your diet habits have been modified by the confinement? (%)	Yes	No		
	38.5	61.5		
With regard to your body weight after the confinement, this was (%)	Increased	Equal	Decreased	Other
	32.4	52.9	14.3	0.4

**Table 6:** Correlations between main variables.

	Time Devoted to Meals	Vegetables Consumption	Fruit Consumption	Legumes Consumption	Sugary Drinks Consumption	Salty Snacks Consumption	Industrial Pastries Consumption
Age	0.114 **	−0.09	−0.020	0.001	−0.047	−0.002	−0.004
Time devoted to meals	1	0.96 *	0.91 *	0.163 **	0.013	0.008	−0.018
Food delivery order at home	−0.013	−0.006	−0.032	−0.055	0.113 **	0.129 **	0.105 *

* *p* < 0.05; ** *p* < 0.01.

## Data Availability

The data presented in this study are available on request from the corresponding authors.

## Supplementary Materials

The following are available online at https://www.mdpi.com/article/10.3390/foods10102443/s1, Table S1: Questionnaire used in the study.
