# Supplementary material for: Has COVID-19 Changed the Lifestyle and Dietary Habits in the Spanish Population after Confinement?
Source: Foods. 2021 Oct 14;10(10):2443. doi: 10.3390/foods10102443 (PMC8535706; doi:10.3390/foods10102443)
Supplement: Supplementary file 1 [file foods-10-02443-s001.zip › foods-1415896-Supplementary.pdf]

Table S1. Questionnaire used in the study

SOCIODEMOGRAPHIC PARAMETERS

Gender

Male

Female

Other

Age

25-34

35-44

45-55

55-64

Place of residence

Living alone

Shared house

Family home

Collective residence

Work type

Presential

Blended job

Unemployed

Student

Other

How many times do you eat per day?

<3

3-5

>5

Which is your mode of eating?

Alone

Shared with family/friends

Watching TV/PC

Others

How many days per week do you order food delivery?

0

1-3

4-6

>6

NUTRITIONAL HABITS

Do you eat bread diary?

Yes

No

If yes, which type of bread?

Wheat bread

Whole wheat bread

Other types

Do you eat oil diary?

Yes

No

Which type of oil?

Olive oil

Sunflower oil

Coconut oil

Vegetable serving (per day)

0

1

2

3

4

5

Fruit serving (per day)

0

1

2

3

4

5

Milk/Dairy serving (per day)

1

2

3

4

5

Dried fruit serving (per day)

0

1

2

3

4

5

Legume serving (per week)

0

1

2

3

4

5

6

7

Potatoes, pasta, rice serving (per week)

0

1

2

3

4

5

6

7

Eggs serving (units/ per week)

0

1

2

3

4

5

6

7

Sugary drinks (units/per week)

0

1

2

3

4

5

6

≥7

Snacks (units/per week)

0

1

2

3

4

5

6

≥7

Industrial pastries (units per week)

0

1

2

3

4

5

6

≥7

FOOD CONSUMPTION

No consumed in a week

Vegetable drink

Red meat

White meat

Sausage meat

White fish

Blue fish

Consumed 1-2 serving/week

Vegetable drinks

Red meat

White meat

Sausage meat

White fish

Blue fish

Consumed ≥ 3 serving/week

Vegetable drinks

Red meat

White meat

Sausage meat

White fish

Blue fish

PHYSICAL ACTIVITY

Do you practice sport?

Yes

No

Where do you practice sport?

Home

Outdoor

Sport centre

How many days practice sport (days/week)?

0

1

2

3

4

5

6

7

Time session (min)

0

<30

30-60

>60

TOXIC HABITS

Do you usually smoke?

Yes

No

Do you increase/decrease smoking after the confinement?

No

Increase

Equal

Decrease

Do you usually drink alcohol?

Yes

No

Do you increase/decrease drinking alcohol after the confinement?

No

Increase

Equal

Decrease

Do you consider that your diet habit has been modified by the confinement?

Yes

No

Have you gained weight during the postconfinement?

Increase

Equal

Decrease

Others

ADHERENCE TO MEDITERRANEAN DIET (0 low; 10 high)

1. Olive oil (≥ 1 spoon/day)

2. Fruit (≥ 1 serving/day)

3. Vegetables or salad (≥ 1 serving/day)

4. Fruit (≥ 1 serving/day) and vegetables (≥1 serving/day)

5. Legumes (≥ 2 servings/week)

6. Fish (≥3 servings/week)

7. Wine (≥1 glass/day)

8. Meat (<1 serving/day)

9. White bread (<1/day) and rice (<1/week)

10. One point is added when ≥ 1 extra serving/day of both fruits and vegetables is consumed
